# Body roundness index outperforms traditional obesity metrics in predicting cardiometabolic risk among children and adolescents: the EMSNGS study

**DOI:** 10.3389/fnut.2026.1760511

**Published:** 2026-02-24

**Authors:** Qifa Hu, Zhongwei Xu, Zhe Su, Zhuoguang Li, Xiu Zhao, Lili Pan, Li Wang

**Affiliations:** 1 Department of Endocrinology, Shenzhen Children's Hospital, Shenzhen, Guangdong, China; 2 Department of Pediatrics, Huidong County People's Hospital, Huizhou, Guangdong, China

**Keywords:** a body shape index, adolescent, body roundness index, cardiometabolic risk index, cardiovascular disease, children

## Abstract

**Objective:**

Novel obesity indices, the body roundness index (BRI) and a body shape index (ABSI), have been proved to be superior over body mass index (BMI) for predicting metabolic syndrome and cardiovascular events in adults. However, their performance in pediatric populations remains unexplored.

**Study design:**

A large-scale cross-sectional study, Evaluation and Monitoring on School-based Nutrition and Growth in Shenzhen (EMSNGS) project, was conducted in 2021 including 4,794 children and adolescents aged 6–17 years. Quantile regression models were used to analyse factors influencing ABSI and BRI. Associations between the 2 novel obesity indices and cardiovascular metabolic risk index (CMRI) were evaluated using logistic regression and receiver operating characteristic (ROC) curves.

**Results:**

In total, 1,971 participants (1,131 boys, 840 girls) exhibited CMRI ≥1. BRI was associated with pubertal development and weight status in both sexes. ABSI was only associated with weight status. BRI was associated with CMRI ≥1, whereas ABSI was not (*P* > 0.05). Compared with waist-to-hip ratio, waist-to-height ratio, and BMI, BRI at the 75th percentile (BRI P75th) demonstrated optimal sensitivity–specificity balance. The area under the ROC curve (95% CI), sensitivity, specificity, and Youden index were 0.752 (0.732–0.771), 71.3%, 79.1%, and 0.504 for boys and 0.693 (0.670–0.716), 61.8%, 76.8%, and 0.386 for girls, respectively.

**Conclusion:**

After adjustment for sex and pubertal stages, BRI P75th remained significantly associated with elevated cardiometabolic risk in children and adolescents, supporting its potential utility as an early screening indicator.

## Introduction

The increasing global prevalence of childhood obesity poses a significant public health challenge. Over the past three decades, China has witnessed a 75.6-fold (1) surge in obesity detection rates among children and adolescents, accompanied by an expanding population base of Pediatric obesity. Concerningly, a substantial proportion (41%−80%) of childhood obesity cases may persist into adulthood, posing significant risks to long-term cardiometabolic health (2). Obesity is a key risk factor for cardiovascular disease (CVD) in adulthood and is strongly associated with early metabolic dysfunction during childhood (3). Although body mass index (BMI) remains the clinical standard for obesity screening, it does not differentiate adipose from lean mass, nor does it quantify fat distribution patterns (4). Individuals with identical BMIs may demonstrate markedly divergent adiposity profiles and body shape (5).

Childhood and adolescence represent critical periods of body composition remodeling, during which significant fluctuations in sex hormone levels play a central role in shaping adipose tissue distribution patterns. The progression of pubertal development (e.g., Tanner staging) and its accompanying hormonal surges constitute the core biological mechanism underlying changes in fat distribution, rather than chronological age by sex (6, 7). Elevated androgen levels promote musculoskeletal development and suppress adipose tissue accumulation. Conversely, estrogen primarily stimulates adipocyte proliferation and activation, thereby promoting subcutaneous fat deposition in women while concurrently inhibiting visceral adipose tissue deposition. Additionally, obesity-induced increases in total adiposity may exacerbate ectopic fat deposition in non-adipose tissues.

Body roundness index (BRI) and a body shape index (ABSI), which integrate height, weight, waist circumference (WC), more accurately reflect body shape characteristics and have garnered increasing attention. BRI employs an elliptical geometric model to quantify trunk fat accumulation, whereas ABSI standardizes waist circumference to circumvent BMI-related confounding. Both indices demonstrate superior predictive capacity over BMI for metabolic syndrome (MetS) and cardiovascular events in adults (8–11). Nevertheless, the relationship between BRI/ABSI and cardiometabolic risk remains poorly defined in Pediatric populations. The most critical underlying reasons include the weight status and puberty-dependent dynamic variability of the BRI/ABSI reference ranges in Pediatric populations and the low incidence rates of cardiovascular events during childhood.

The cardiometabolic risk index (CMRI), a continuous composite metric integrating multidimensional biomarkers (e.g., blood pressure, lipid profiles, and fasting glucose), demonstrates superior sensitivity compared to traditional categorical definitions of MetS (12, 13). Consequently, CMRI may serve as a more robust tool for predicting cardiovascular risk trajectories in pediatric populations. The relationship between these novel adiposity indices (BRI and ABSI) and cardiometabolic risk profiles remains uncharted in Pediatric research. Using data from the Evaluation and Monitoring on School-based Nutrition and Growth in Shenzhen (EMSNGS) study, the present study aimed to elucidate the relationships among BRI, ABSI, and CMRI in children and adolescents based on established weight status-, sex-, and puberty stage-specific references and compare the predictive efficacy of these novel indices against conventional obesity parameters.

## Methods

### Participants

This study utilized data from the EMSNGS project (Chinese Clinical Trial Registry: ChiCTR2100051722). This large-scale longitudinal study was initiated in 2021 by Shenzhen Children's Hospital and Shenzhen Chronic Disease Control Center. Employing random stratified sampling, the project included one elementary school, one junior high school, and one senior high school from each of Shenzhen's 10 districts. A total of 27 schools were sampled, including three institutions encompassing both elementary and junior high divisions. One class was randomly selected from each school per grade level, excluding classes with fewer than 40 students. The final sample comprised 112 classes with 4,794 participants who completed comprehensive physical examinations and medical assessments.

**Figure 1:**
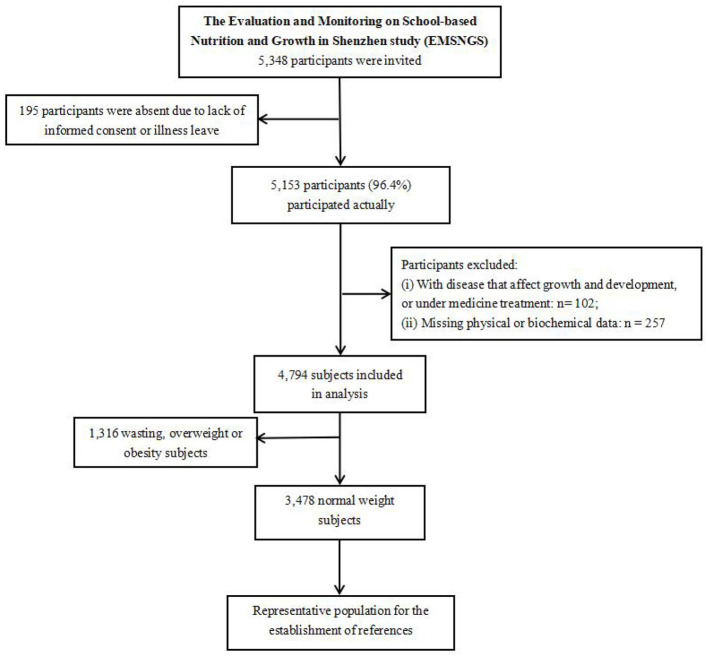
Flowchart of the selected study population.

A schematic representation of the overall study design is shown in Figure 1. The inclusion criteria were: ① participation in the current epidemiological survey with signed informed consent; ② age 6–17 years (under 18 years old on the physical examination date); and ③ availability of complete study-related data.

The exclusion criteria were: ① individuals with severe organ dysfunction, endocrine/metabolic disorders (e.g., growth hormone deficiency, thyroid dysfunction, hematologic/oncologic diseases, chronic hepatic/renal diseases); ② prolonged use of medications or supplements known to significantly affect growth or metabolism (e.g., growth hormone, glucocorticoids, immunosuppressants, weight-altering supplements); ③ physical disabilities or missing anthropometric data (height, weight, and WC); ④ incomplete demographic data (age, sex); ⑤ sexual developmental abnormalities: precocious puberty (secondary sexual characteristics appearing before age 8 in girls or 9 in boys; menarche <10 years), or delayed puberty (absence of secondary sexual characteristics by age 13 in girls or 14 in boys); and, ⑥ psychomotor developmental delay.

Written informed consent was obtained from legal guardians, and additional independent consent was obtained from high school-aged adolescents. Ethical approval was granted by the Shenzhen Ethics Committee (Approval No. 2021105). This study was registered in the Chinese Clinical Trial Registry (registration no. ChiCTR2100051722) and strictly adhered to the Declaration of Helsinki.

### Anthropometric measurement

Trained project investigators conducted standardized anthropometric and developmental assessments using uniform measuring instruments. Data collected included height, weight, WC, and pubertal development indicators (e.g., testicular volume in boys and breast development stages in girls).

Height and weight measurement: Height and weight were measured using the Shanghe Ultrasonic Height-Weight Meter (SH-200, Zhengzhou Shanghe electronic Technology company, Zhengzhou City, China), with a precision of 0.1 cm and 0.1 kg, respectively.

Waist circumference (WC) measurement: A standardized leather circumference tape (National Institute of Sports Science) with a maximum range of 200 cm and accuracy of 0.1 cm was used. The tape was positioned horizontally at the midpoint between the 12th rib at the mid-axillary line and the highest point of the iliac crest. Measurements were taken at the end of normal expiration and recorded in centimeters (accurate to 0.1 cm). Two measurements were performed; if the difference exceeded 1 cm, a third measurement was performed. The average of the two closest values was calculated. The leather tape was regularly calibrated before and during the survey against a non-stretch steel ruler and was replaced with a new one after every 50 participants to minimize potential stretching.

Hip circumference (HC) measurement: The same leather tape was applied horizontally around the most prominent posterior point on the buttocks. Measurements were recorded at the end of normal expiration (accurate to 0.1 cm), following the same two-measurement protocol as that for WC.

Assessment of pubertal development: All examiners received standardized training from the Endocrinology Department of Shenzhen Children's Hospital. The measurement process was strictly supervised on-site by Pediatric endocrinologists at the same institution. Pubertal stages were evaluated according to the Tanner criteria. In girls, the breast development stage (B1–B5) was determined through visual inspection and palpation. Bilateral assessments were recorded, with the more advanced stages used for the final classification. In boys, testicular volume (mL) was measured by palpation and compared using a Prader orchidometer. The larger volume was selected if asymmetry existed. Additionally, the participants were interviewed regarding pubertal milestones, which comprised menarche status and age at menarche (if applicable) in girls and spermarche status and age at spermarche (if applicable) in boys.

Blood pressure measurement: Blood pressure was measured by using an upper-arm electronic sphygmomanometer (Omron HEM1320, Omron, Dalian, China). Before measurement, the subjects were instructed to rest quietly for 15–30 min in a temperature-controlled, quiet environment; avoid strenuous activity; and empty their bladders. Two consecutive measurements were recorded. If the difference between two readings exceeded 5 mmHg, or above the hypertension cutoff value (P95th for age, sex, height), additional measurements were performed until consistency (within 5 mmHg) was achieved.

### Biochemical measurements

All study participants fasted for 10 h (no food or water intake) before venous blood collection in the morning. Serum samples were separated by centrifugation and analyzed using a fully automated biochemical analyser (Beckman Coulter AU5811). Fasting blood glucose (FBG), high-density lipoprotein cholesterol (HDL-C), and triglyceride (TG) levels were measured.

### Diagnostic criteria

Assessment of weight status: BMI was calculated as weight in kilograms divided by height in meters squared (kg/m^2^). Weight status was categorized into four groups based on sex- and age-specific BMI percentiles (14, 15): wasting: BMI <5th percentile for sex and age; normal weight: BMI ≥5th percentile but <85th percentile for sex and age; overweight: BMI ≥85th percentile but <95th percentile for sex and age; and, obesity: BMI ≥95th percentile for sex and age.

Pubertal Staging: Pubertal development was assessed using the Tanner staging system. Breast development stage was documented in girls, while in boys, testicular volume (mL) was measured via palpation using a Prader orchidometer. Based on secondary sexual characteristics, participants were categorized into three groups: prepubertal stage (Tanner stage I), early-middle pubertal stage (Tanner stages II-III), or late pubertal stage (Tanner stages IV-V). The prepubertal stage (Tanner I) was defined as the absence of signs of sexual maturation in both sexes, while the pubertal stage (Tanner II-V) was defined as: testicular volume ≥4 mL or spermarche in boys, and breast development ≥ stage B2 or post-menarche in girls (16, 17).

Definition of cardiometabolic risk: Cardiometabolic risk was defined using the CMRI which defined as follows: CMRI z-score = WC z-score + SBP z-score + DBP z-score + GLU z-score + TG z-score – HDL-c z-score. In this study, sex- and age-specific means and standard deviations (SD) for each CMRI component were established using a reference population with BMI between the 5th and 95th percentiles. Age- and sex-adjusted CMRI values were computed based on these standardized parameters. Higher CMRI values indicated an elevated cardiometabolic risk. Increasing CMRI values indicated heightened cardiometabolic risk, using z-score ≥1 as the threshold for high risk. The threshold of CMRI z-score ≥1 was chosen to define “high cardiometabolic risk” (18–20) as it represents a value one standard deviation above the mean of the reference population (children with normal BMI). This cutoff is statistically conventional for identifying individuals at increased risk within a normally distributed continuous variable and has been employed in prior studies to define elevated cardiometabolic risk clusters.

The calculation formulas of these anthropometric indices:

BMI: Weight (kg)/Height (m^2^).

Waist-to-Height Ratio (WHtR): WC (cm)/height (cm).

Waist-to-Hip Ratio (WHR): waist circumference (cm)/hip circumference (cm).

ABSI (7): WC (m)/(BMI (kg/m^2^)^2/3^ × height (m)^1/2^).

BRI (6): 364.2 – 365.5 × (1– (WC (m)/2π)^2/^(0.5 × Height (m)^2^)^1/2^.

### Quality control protocol

All measurements were conducted by a unified survey team following standardized training. All instruments were calibrated prior to testing, and random quality checks were performed during data collection. Post-survey data verification was conducted daily with immediate retesting for outliers. A centralized database was developed by the Shenzhen Chronic Disease Control Center (SCCDC). District-level teams completed the data entry, followed by cross-validation and feedback from the SCCDC. Final data reconciliation was performed by the district-level chronic disease agencies. All data underwent an independent double-entry verification protocol, with dual researchers crosschecking and inputting the records, followed by database construction using EpiData software (version 3.0; EpiData Association, Odense, Denmark).

### Statistical analysis

Data were analyzed using SPSS version 26.0 (IBM, Armonk, NY, USA). Normally distributed continuous variables were expressed as mean ± standard deviation (SD), whereas non-normally distributed variables were presented as median and interquartile range. Independent sample *t*-tests were used for normally distributed data, non-parametric tests for skewed data, and chi-square tests for categorical comparisons. Sex-specific determinants of ABSI and BRI were analyzed using quantile regression. Quantile regression models were applied at the 5th, 25th, 50th, 75th, and 95th percentiles to capture the full distribution of BRI and ABSI. Covariates including pubertal stage and weight status were adjusted based on prior evidence of their confounding effects. Sex-specific percentile references for the BRI and ABSI were established based on the quantile regression models. Participants were categorized into four groups according to BRI, ABSI, WHtR, WHR, and BMI quartiles (Q1 < 25th, 25th ≤ Q2 < 50th, 50th ≤ Q3 < 75th, and Q4 ≥ 75th). Multivariate binary logistic regression was employed to calculate odds ratios (ORs) for the association of BRI, ABSI, WHtR, WHR, and BMI with high cardiometabolic risk (CMRI ≥1). The second quantile (Q2) was chosen as the reference group for logistic regression analyses because the first quantile (Q1) may include individuals with underweight or atypical body composition, who are not an ideal “low-risk” reference group for cardiometabolic outcomes. The diagnostic performance of novel obesity indices (BRI, ABSI) in identifying high cardiometabolic risk (CMRI ≥1) was evaluated using receiver operating characteristic (ROC) curve analysis. The optimal cutoff values were determined by maximizing the area under the curve (AUC), sensitivity, and specificity (i.e. the highest Youden index).

## Results

### General characteristics

The study initially sampled 5,348 individuals, of whom 5,184 participated in the survey (consent rate: 96.9%). Valid responses were obtained from 5,153 participants (response rate: 96.4%); ultimately, 4,794 participants were included in the analysis (2,656 boys and 2,138 girls). Of these, 1,971 participants (1,131 boys and 840 girls) had a CMRI ≥1 (Table 1). The proportion of boys and girls with high CMRI scores gradually increased with the progression of BMI and pubertal development (Table 2).

**Table 1:** General characteristics of the participants.

**Variables**	**Boys**	**Girls**
Total number	2,656 (55.4%)	2,138 (44.6%)
**Age**		
6–8 years	591 (22.3%)	487 (22.8%)
9–11 years	707 (26.6%)	597 (27.9%)
12–14 years	705 (26.5%)	576 (26.9%)
15–17 years	653 (24.6%)	478 (22.4%)
**Pubertal staging**		
Prepubertal	951 (35.8%)	544 (25.4%)
Pubertal	1,705 (64.2%)	1,594 (74.6%)
**Weight status**		
Wasting	117 (4.4%)	68 (3.2%)
Normal weight	1,862 (70.1%)	1,616 (75.6%)
Overweight	319 (12.0%)	233 (10.9%)
Obesity	358 (13.5%)	221 (10.3%)
**High CMRI**		
CMRI ≥ 1	1,131 (42.6%)	840 (39.3%)

CMRI, cardiometabolic risk index. High CMRI was defined as CMRI ≥ 1.

**Table 2:** Distribution of high CMRI in children and adolescents.

**Variables**	**Total**	**Boys**	**Girls**
Prevalence of high CMRI	1,971 (41.1%)	1,131 (42.6%)	840 (39.3%)
**Weight status**			
Wasting (*n* = 185)	12 (6.5%)	3 (2.6%)	9 (13.2%)
Normal weight (*n* = 3,478)	1,004 (28.9%)	538 (28.9%)	466 (28.8%)
Overweight (*n* = 552)	410 (74.3%)	250 (78.4%)	160 (68.7%)
Obesity (*n* = 579)	545 (94.1%)	340 (95.0%)	205 (92.8%)
**Age**			
6–8 years (*n* = 1,078)	397 (36.8%)	235 (39.8%)	162 (33.3%)
9–11 years (*n* = 1,304)	570 (43.7%)	312 (44.1%)	258 (43.2%)
12–14 years (*n* = 1,281)	547 (42.7%)	304 (43.1%)	243 (42.2%)
15–17 years (*n* = 1,131)	457 (40.4%)	280 (42.9%)	177 (37.0%)
**Pubertal staging**			
Prepubertal (*n* = 1,495)	530 (35.5%)	373 (39.2%)	157 (28.9%)
Pubertal (*n* = 3,299)	1,441 (43.7%)	758 (44.5%)	683 (42.8%)

CMRI, cardiometabolic risk index. High CMRI defined as CMRI ≥ 1.

### The relationship among pubertal stage, weight status, and BRI/ABSI

Multivariate quantile regression analyses revealed significant associations between BRI and pubertal staging/weight status in girls and boys, whereas the ABSI showed correlations exclusively with weight status (Tables 3–**6**).

**Table 3:** Multivariate quantile regression analysis between pubertal stage and weight status and BRI in girls.

**Variables**	**BRI**				
	**P5th**	**P25th**	**P50th**	**P75th**	**P95th**
**Pubertal staging**					
Prepubertal	Reference				
Pubertal	−0.083 (−0.151 to −0.014)^*^	−0.073 (−0.128 to −0.019)^*^	−0.026 (−0.085 to 0.034)^*^	0.029 (−0.045 to 0.102)	0.106 (−0.151 to −0.014)
**Weight status**					
Wasting	Reference				
Normal weight	0.357 (0.188–0.526)^**^	0.395 (0.261–0.530)^**^	0.517 (0.369–0.665)^**^	0.625 (0.444–0.806)^**^	0.746 (0.188–0.526)^**^
Overweight	1.082 (0.893–1.271)^**^	1.144 (0.993–1.294)^**^	1.338 (1.173–1.503)^**^	1.459 (1.257–1.662)^**^	1.552 (0.893–1.271)^**^
Obesity	1.567 (1.377–1.757)^**^	1.829 (1.678–1.980)^**^	2.222 (2.056–2.388)^**^	2.587 (2.383–2.790)^**^	2.872 (1.377–1.757)^**^

The values outside the brackets in the table are the β coefficients of quantile regression, and the values inside the brackets are 95% confidence intervals. BRI, body roundness index.

^*^P < 0.05; ^**^P < 0.001.

Quintile regression analysis (BRI): For girls, the regression coefficients for normal weight (P5th–P95th), overweight (P5th–P95th), obesity (P5th–P95th), and puberty (P5th–P50th) were statistically significant (*P* < 0.05; Table 3). For boys, the regression coefficients for normal weight (P5th–P95th), overweight (P5th–P95th), obesity (P5th–P95th), and puberty (P5th–P75th) were significant (Table 4).

**Table 4:** Multivariate quantile regression analysis between pubertal stage and weight status and BRI in boys.

**Variables**	**BRI**				
	**P5th**	**P25th**	**P50th**	**P75th**	**P95th**
**Pubertal staging**					
Prepubertal	Reference				
Pubertal	−0.2 (−0.241 to −0.115)^*^	−0.211 (−0.258 to −0.164)^*^	−0.166 (−0.215 to −0.118)^*^	−0.086 (−0.148 to −0.023)^*^	0.054 (−0.06 to −0.168)
**Weight status**					
Wasting	Reference				
Normal weight	0.387 (0.239 to 0.543)^**^	0.472 (0.362 to 0.582)^**^	0.515 (0.401 to 0.628)^**^	0.696 (0.549 to 0.844)^**^	0.893 (0.624 to 1.161)^**^
Overweight	1.292 (1.125 to 1.459)^**^	1.509 (1.384 to 1.633)^**^	1.619 (1.490 to 1.747)^**^	1.797 (1.629 to 1.964)^**^	1.955 (1.650 to 2.259)^**^
Obesity	2.071 (1.906 to 2.235)^**^	2.348 (2.226 to 2.471)^**^	2.619 (2.493 to 2.746)^**^	3.042 (2.878 to 3.207)^**^	3.670 (3.371 to 3.970)^**^

The values outside the brackets in the table are the β coefficients of quantile regression, and the values inside the brackets are 95% confidence intervals. BRI, body roundness index.

^*^P < 0.05; ^**^P < 0.001.

Quintile regression analysis (ABSI): In girls, significant associations were observed across multiple categories: normal weight (P5th–P95th), overweight (P5th–P95th), obesity (P5th), and puberty (P5th–P25th, P95th) (Table 5). For boys, the regression coefficients for normal-weight (P5th–P75th), overweight (P5th–P25th), and obesity (P50th–P75th) were statistically significant (Table 6).

**Table 5:** Multivariate quantile regression analysis between pubertal stage and weight status and ABSI in girls.

**Variables**	**ABSI**				
	**P5th**	**P25th**	**P50th**	**P75th**	**P95th**
**Pubertal staging**					
Prepubertal	Reference				
Pubertal	−1.3 (−2.07 to −0.57)^**^	−0.9 (−1.42 to −0.41)^**^	−0.4 (−0.89 to 0.08)	0.2 (−0.29 to 1.03)	1.3 (0.09 to 2.48)^*^
**Weight status**					
Wasting	Reference				
Normal weight	−2.5 (−4.4 to −0.68)^**^	−1.6 (−2.84 to −0.36)^*^	−1.8 (−3.0 to −0.59)^**^	−2.0 (−3.65 to −0.37)^**^	−4.0 (−6.96 to −1.05)^**^
Overweight	−3.5 (−5.54 to −1.38)^**^	−2.0 (−3.43 to −0.65)^**^	−2.3 (−3.66 to −0.97)^**^	−2.3 (−4.15 to −0.49)^*^	−4.0 (−7.23 to −0.69)^*^
Obesity	−2.8 (−4.87 to −0.68)^**^	−0.9 (−2.27 to 0.52)	−1.0 (−2.35 to 0.36)	0 (−1.89 to 1.80)	−3.3 (−6.60 to 0.04)

The values outside the brackets in the table are the β coefficients of quantile regression, and the values inside the brackets are 95% confidence intervals. BRI, body roundness index. ^*^P < 0.05; ^**^P < 0.001. ABSI values were scaled by a factor of 1,000 for regression analysis to yield interpretable coefficients.

**Table 6:** Multivariate quantile regression analysis between pubertal stage and weight status and ABSI in boys.

**Variables**	**ABSI**				
	**P5th**	**P25th**	**P50th**	**P75th**	**P95th**
**Pubertal staging**					
Prepubertal	Reference				
Pubertal	0 (−0.56 to −0.56)	−0.3 (−0.67 to −0.07)	−0.2 (−0.59 to 0.16)	0 (−0.4 to 0.49)	0.8 (−0.14 to 1.76)
**Weight status**					
Wasting	Reference				
Normal weight	−1.8 (−3.14 to −0.51)^**^	−2.5 (−3.36 to −1.61)^**^	−1.8 (−2.71 to −0.95)^**^	−1.2 (−2.29 to−0.20)^*^	−0.8 (−3.03 to 1.43)
Overweight	−1.8 (−3.34 to−0.36)^*^	−1.8 (−2.77 to −0.78)^**^	−0.3 (−1.27 to 0.73)	1.0 (−0.19 to 2.18)	0.8 (−1.70 to 3.37)
Obesity	0 (−1.42 to 1.52)	0.1 (−0.92 to 1.04)	1.6 (0.61 to 2.57)^**^	2.5 (1.34 to 3.68)^**^	1.4 (−1.14 to 3.85)

The values outside the brackets in the table are the β coefficients of quantile regression, and the values inside the brackets are 95% confidence intervals. BRI, body roundness index. ^*^P < 0.05; ^**^P < 0.001. ABSI values were scaled by a factor of 1,000 for regression analysis to yield interpretable coefficients.

### Establishment of BRI and ABSI percentile references based on quantile regression

Pediatric reference percentiles for BRI/ABSI were derived from quantile regression analyses of local normal-weight cohorts segregated by sex.

Sex-differentiated BRI percentile norms were established by segmenting the male and female cohorts by pubertal developmental phase (prepubertal/pubertal) (Tables 7, 8) based on a normal-weight cohort.

**Table 7:** Percentile reference values for BRI and ABSI in girls.

**Variables**	**P5th**	**P25th**	**P50th**	**P75th**	**P95th**
**BRI**					
Prepubertal (*n* = 442)	1.156	1.459	1.709	1.985	2.509
Pubertal (*n* = 1,174)	1.084	1.398	1.677	2.025	2.592
**ABSI**					
Normal weight (*n* = 1,616)	0.0727	0.0762	0.0788	0.0815	0.0863

BRI, body roundness index; ABSI, A body shape index.

**Table 8:** Percentile reference values for BRI and ABSI in boys.

**Variables**	**P5th**	**P25th**	**P50th**	**P75th**	**P95th**
**BRI**					
Prepubertal (*n* = 687)	1.228	1.586	1.816	2.115	2.605
Pubertal (*n* = 1,175)	1.039	1.348	1.645	2.038	2.670
**ABSI**					
Normal weight (*n* = 1,862)	0.0734	0.0764	0.0787	0.0811	0.0860

BRI, body roundness index; ABSI, A body shape index.

Sex-differentiated ABSI percentile norms were established by analyzing male and female cohorts separately. The ABSI standard was constructed based only on the normal-weight cohort (Tables 7, 8).

### Binary logistic regression analysis of BRI and ABSI in relation to high cardiometabolic risk

**Figure 2:**
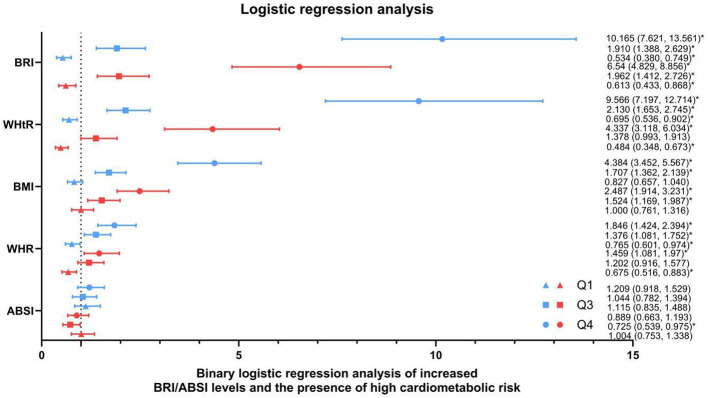
Binary logistic regression analysis of high BRI/ABSI levels and the presence of high cardiometabolic risk. Logistic regression analysis was used to calculate the odds ratios of high CMRI associated with the increase in the BRI, WHtR, BMI, WHR, and ABSI, compared to the second quantile group (Q2) and are presented in the figure, where blue lines represent boys and red lines represent girls. Q1 to Q4 represent the quartiles of grip strength indices: quartiles (Q1 < 25th, 25th ≤ Q2 < 50th, 50th ≤ Q3 < 75th, and Q4 ≥ 75th). High CMRI was defined as CMRI ≥ 1. All estimates are adjusted for age and pubertal stage. CMRI, cardiometabolic risk index; BMI, body mass index; WHR, waist-to-hip ratio; WHtR, waist-to-height ratio; ABSI, A body shape index; BRI, body roundness index. ^*^P < 0.05.

Using the presence of high cardiometabolic risk (defined as CMRI ≥1) as the dependent variable, novel obesity indices (BRI and ABSI) were analyzed as independent variables, and the participants categorized into quantile groups, with the second quantile group (Q2) serving as the reference. Girls and boys in the fourth BRI quantile group (Q4) exhibited significantly elevated cardiometabolic risk, with adjusted ORs of 6.540 (95% CI: 4.829–8.856) and 10.165 (95% CI: 7.621–13.561) compared to Q2, respectively. In contrast, ABSI showed no statistically significant association with CMRI ≥1 in either sex (*P* > 0.05). With the increase in the WHtR, WHR, and BMI, the presence of high cardiometabolic risk gradually increased (Figure 2).

### ROC curve analysis

**Figure 3:**
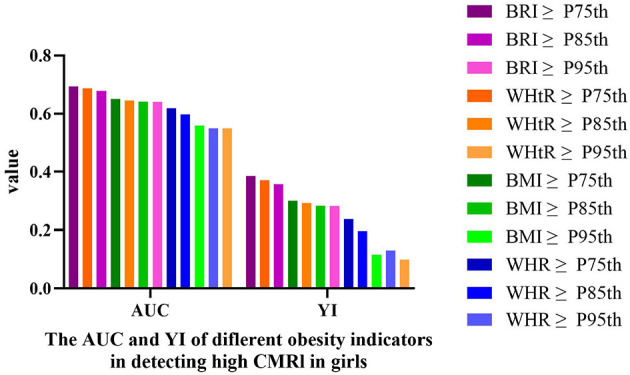
The AUC and YI of different obesity indicators in detecting high CMRI in girls. High CMRI defined as CMRI ≥ 1. AUC, area under the curve; YI, Youden index; CMRI, cardiometabolic risk index; WHR, waist-to-hip ratio; WHtR, waist-to-height ratio; BRI, body roundness index; BMI, body mass index.

**Figure 4:**
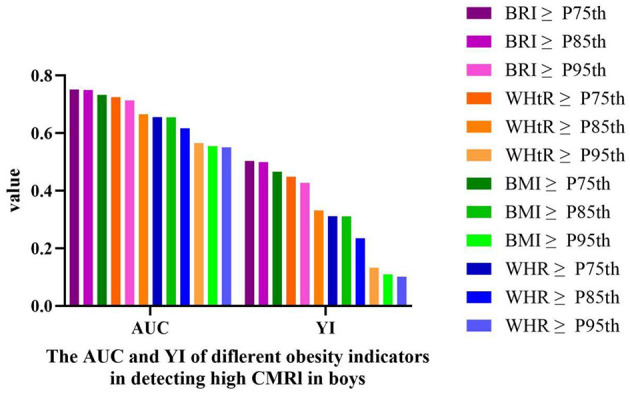
The AUC and YI of different obesity indicators in detecting high CMRI in boys. High CMRI defined as CMRI ≥ 1. AUC, area under the curve; YI, Youden index; CMRI, cardiometabolic risk index; WHR, waist-to-hip ratio; WHtR, waist-to-height ratio; BRI, body roundness index; BMI, body mass index.

The ROC curve analysis comparing obesity indices for detecting high cardiometabolic risk (CMRI ≥1) revealed that the 75th percentile of BRI demonstrated optimal diagnostic performance in both sexes. Compared to sex- and age-matched thresholds of WHR (WHR: P75th/P85th/P95th), WHtR (WHtR: P75th/P85th/P95th), and BMI (BMI: P75th/P85th/P95th), BRI-P75th achieved the highest Youden index (girls: AUC = 0.693 [95% CI: 0.670–0.716], sensitivity = 61.80%, specificity = 76.80%, Youden = 0.386, Table 9 and Figure 3; boys: AUC = 0.752 [95% CI: 0.732–0.771], sensitivity = 71.3%, specificity = 79.1%, Youden = 0.504, Table 10 and Figure 4).

**Table 9:** The sensitivity, specificity, and AUC of different obesity indicators in detecting high CMRI in girls.

**Variables**	**AUC**	**Sensitivity (%)**	**Specificity (%)**	***P-value***	**95%CI**		**YI**
BRI ≥ P75th	0.693	61.8	76.8	<0.01	0.670	0.716	0.386
BRI ≥ P85th	0.678	50.7	85.0	<0.01	0.654	0.703	0.357
BRI ≥ P95th	0.641	35.0	93.3	<0.01	0.617	0.666	0.283
WHR ≥ P75th	0.619	43.8	80.0	<0.01	0.594	0.644	0.238
WHR ≥ P85th	0.598	28.9	90.8	<0.01	0.573	0.624	0.197
WHR ≥ P95th	0.549	11.5	98.3	<0.01	0.524	0.575	0.098
WHtR ≥ P75th	0.686	51.3	85.8	<0.01	0.662	0.710	0.371
WHtR ≥ P85th	0.646	34.2	95.1	<0.01	0.621	0.671	0.293
WHtR ≥ P95th	0.549	13.3	99.7	<0.01	0.540	0.591	0.130
BMI ≥ P75th	0.651	35.2	94.9	<0.01	0.624	0.677	0.301
BMI ≥ P85th	0.642	32.1	96.3	<0.01	0.617	0.667	0.284
BMI ≥ P95th	0.558	11.7	99.8	<0.01	0.532	0.583	0.115

CMRI, cardiometabolic risk index. High CMRI defined as CMRI ≥ 1. AUC, area under the curve; CI, confidence interval; YI, Youden index; WHR, waist-to-hip ratio; WHtR, waist-to-height ratio; BRI, body roundness index; BMI, body mass index.

**Table 10:** The sensitivity, specificity, and AUC of different obesity indicators in detecting high CMRI in boys.

**Variables**	**AUC**	**Sensitivity (%)**	**Specificity (%)**	***P-value***	**95%CI**		**YI**
BRI ≥ P75th	0.752	71.3	79.1	<0.01	0.732	0.771	0.504
BRI ≥ P85th	0.750	62.9	87.1	<0.01	0.730	0.769	0.500
BRI ≥ P95th	0.714	48.4	94.4	<0.01	0.693	0.734	0.428
WHR ≥ P75th	0.656	45.0	86.2	<0.01	0.634	0.677	0.312
WHR ≥ P85th	0.617	29.9	93.6	<0.01	0.595	0.639	0.235
WHR ≥P95th	0.551	11.7	98.5	<0.01	0.528	0.573	0.102
WHtR ≥ P75th	0.725	53.2	91.7	<0.01	0.704	0.745	0.449
WHtR ≥ P85th	0.666	35.8	97.4	<0.01	0.644	0.688	0.332
WHtR ≥ P95th	0.566	13.4	99.9	<0.01	0.544	0.588	0.133
BMI ≥ P75th	0.733	57.3	89.4	<0.01	0.713	0.753	0.466
BMI ≥ P85th	0.655	32.8	98.3	<0.01	0.634	0.677	0.311
BMI ≥ P95th	0.555	11.1	99.9	<0.01	0.533	0.577	0.110

CMRI, cardiometabolic risk index. High CMRI defined as CMRI ≥ 1. AUC, area under the curve; CI: confidence interval; YI, Youden index; WHR, waist-to-hip ratio; WHtR, waist-to-height ratio; BRI, body roundness index; BMI, body mass index.

## Discussion

This study utilized data from the EMSNGS project to delineate the relationships between BRI and pubertal stage and weight status among children and adolescents aged 6–17 years. For the first time, normative reference values for BRI were constructed based on sex and pubertal stage while evaluating the associations of novel obesity indices, i.e., BRI and ABSI, —with cardiometabolic risk (CMRI ≥ 1) compared to conventional obesity metrics (BMI, WHR, and WHtR). BRI was significantly superior in predicting elevated cardiometabolic risk, whereas ABSI showed no significant association. These findings provide critical evidence for early screening of obesity-related metabolic risks in the Pediatric population.

The study revealed a robust positive correlation between BRI and high cardiometabolic risk (CMRI ≥ 1). Among girls and boys, the highest BRI quartile (Q4) conferred 6.540-fold (95% CI: 4.829–8.856) and 10.165-fold (95% CI: 7.621–13.561) higher risks compared to the second quartile (Q2), respectively. In the ROC curve analysis, the 75th percentile threshold of BRI demonstrated an optimal sensitivity–specificity balance for predicting high CMRI (boys: AUC = 0.752, sensitivity 71.3%, specificity 79.1%; girls: AUC = 0.693, sensitivity 61.80%, specificity 76.80%), outperforming BMI, WHR, and WHtR. Further, BRI, which integrates waist circumference and height, more accurately reflects abdominal adiposity and its metabolic sequelae. Although research in adults highlights BRI's superiority in predicting cardiovascular and diabetic risks (21, 22), Pediatric evidence remains inconsistent. For instance, Chen et al. (23) reported BRI's enhanced discriminative capacity for dyslipidaemia and hypertension over BMI and ABSI in children aged 7–17 years, whereas Cristine Silva et al. (6) found that BRI was no better than conventional indices. These discrepancies may stem from varying diagnostic criteria for hypertension/dyslipidaemia, population-specific fat distribution patterns, or metabolic heterogeneity. In addition, the reference range of BRI P5th–P95th for adolescent girls and boys was 1.084–2.592 and 1.039–2.670, respectively, which differed markedly from the adult BRI reference range of 4.5–5.5 (11). This discrepancy likely reflects differences in the study endpoints. Thus, large-scale longitudinal studies are warranted to validate the clinical utility of BRI in Pediatric cohorts.

BRI, grounded in elliptical geometry and derived from WC and height, addresses BMI's inability to differentiate fat and lean mass, better capturing the pathophysiological link between abdominal adiposity and metabolic dysfunction (8). In contrast, ABSI showed no significant association with CMRI ≥ 1 in the logistic regression analysis (*P* > 0.05). Although the ABSI aims to standardize waist circumference while mitigating BMI-related confounding, its limited predictive value for Pediatric metabolic risk may reflect its failure to account for visceral fat dynamics or metabolic adaptations (9). Although ABSI modeling standardizes WC for the effects of BMI and height, ABSI was developed based on body morphology within the US population. It cannot account for racial heterogeneity and is therefore not universally applicable across all populations. In a study by Rahimi et al., the predictive performance of the ABSI, BMI, WC, BRI, and WHtR for MetS in adults demonstrated that WHtR and BRI were the strongest predictors of MetS. Conversely, ABSI showed the weakest correlation with metabolic variables (24). Intriguingly, quantile regression revealed that ABSI was significantly associated with obesity in boys at higher percentiles (β = 0.002–0.003, *P* < 0.001; Table 6), suggesting potential context-specific utility warranting further investigation.

Recent studies underscore sex- and puberty-dependent trajectories of body fat distribution in children and adolescents aged 3–17 (6). Our quantile regression analyses further delineated these relationships. In boys, BRI correlated significantly with pubertal stage (β = −0.086 to −0.211, *P* < 0.001; Table 4) and weight status across all percentiles (β = 0.387–3.670, *P* < 0.001). In girls, BRI was strongly associated with weight status (β = 0.357–2.872, *P* < 0.001; Table 3) but only weakly with pubertal staging at lower percentiles (P5th–P25th). Consistent with our findings, Chen et al. (22) reported significantly lower BRI values in 7–17-year-old adolescents (pubertal stage) than in 3–6-year-old children (prepubertal stage) in a study of 1,587 children and adolescents. However, the study did not incorporate a physical examination of secondary sexual characteristics, rendering its pubertal staging method potentially inaccurate. Given that the 7–17-year age group may include prepubertal children, these conclusions are subject to limitations. In contrast, our study utilized the Tanner staging system to precisely define pubertal status and employed quantile regression analysis, demonstrating that the BRI exhibited a significant inverse correlation with pubertal development; that is, BRI values were substantially lower during puberty than during the prepubertal stage. This divergence may be explained by sex-specific changes in body composition during puberty. In boys, the testosterone surge promotes a marked increase in lean mass, which may disproportionately increase relative to central fat mass, leading to a decrease in BRI. In girls, estrogen promotes fat accumulation preferentially in gluteofemoral rather than abdominal depots. Since BRI primarily reflects abdominal circumference, the pubertal increase in overall adiposity in girls may be partially offset by this distribution pattern, resulting in a less pronounced pubertal change in BRI compared to boys (24, 25). In girls, estrogen enhances subcutaneous fat expandability via estrogen receptor signaling while suppressing visceral adipogenesis (26), thereby minimizing pubertal variation in the BRI. Additionally, heightened dietary awareness and body image concerns among girls may explain their greater sensitivity to weight status (27). Based on these findings, we established sex- and puberty-specific BRI percentile references for boys and girls (Tables 7, 8).

This is the first study to develop sex-, weight status-, and puberty-specific BRI reference values for children and adolescents. This study analyzed the impact of puberty and weight status on BRI across sexes using quantile regression methods. However, the sample was drawn exclusively from an urban population in Shenzhen. Although rigorous sampling was employed, the findings may not be fully generalizable to rural populations or other regions with different ethnic, socioeconomic, or lifestyle characteristics, which limits the external validity of our proposed reference percentiles and cut-offs. Although calibrated, the use of a leather tape for waist circumference measurements, as opposed to an inelastic tape, may introduce a small systematic error. Moreover, residual confounding effects from unmeasured key modifiable lifestyle factors such as detailed dietary intake and objective physical activity levels, along with the cross-sectional design, limit causal inference and long-term assessment of cardiometabolic risks. A methodological consideration is that waist circumference is a component of both BRI and the CMRI. This partial mathematical overlap may inflate the strength of the association (e.g., the high odds ratios) between BRI and high CMRI. While this does not invalidate the observed correlation, it suggests that the predictive performance of BRI should be interpreted with this coupling in mind. Using the same approach, we also analyzed the association between CMRI (after omitting WC) and BRI. Although the odds ratio (OR) was attenuated relative to previous estimates, the adjusted ORs remained significantly elevated—specifically, 3,288 for boys and 2,651 for girls. The results consistently indicated a strong correlation between BRI and CMRI (Supplementary Table 1). Our study suggests that the BRI, particularly its 75th percentile cut-off, holds potential as a simple, non-invasive tool for early identification of children and adolescents at higher cardiometabolic risk within clinical or school health settings. Future large-scale, longitudinal studies are needed to validate the predictive value of BRI for cardiometabolic endpoints and to establish definitive, population-specific reference standards. Further research should also investigate the interplay between BRI, lifestyle factors (e.g., diet, physical activity), and genetic predispositions to better understand and mitigate cardiometabolic risk from childhood.

## Conclusion

When adjusted for sex and pubertal developmental stages, the body roundness index at the 75th percentile remained significantly associated with elevated cardiometabolic risk (CMRI ≥1) in children and adolescents, indicating its potential as an early screening indicator for cardiovascular disease (CVD) risk.

## Data Availability

The original contributions presented in the study are included in the article/Supplementary material, further inquiries can be directed to the corresponding author.
