# Correction: Body roundness index outperforms traditional obesity metrics in predicting cardiometabolic risk among children and adolescents: the EMSNGS study

**DOI:** 10.3389/fnut.2026.1821863

**Published:** 2026-03-26

**Authors:** Qifa Hu, Zhongwei Xu, Zhe Su, Zhuoguang Li, Xiu Zhao, Lili Pan, Li Wang

**Affiliations:** 1 Department of Endocrinology, Shenzhen Children's Hospital, Shenzhen, Guangdong, China; 2 Department of Pediatrics, Huidong County People's Hospital, Huizhou, Guangdong, China

**Keywords:** a body shape index, adolescent, body roundness index, cardiometabolic risk index, cardiovascular disease, children

The funder “Sanming Project of Medicine in Shenzhen, No.202411011” to author Zhe Su was erroneously omitted.

The correct **Funding** statement is as follows:

“The author(s) declared that financial support was received for this work and/or its publication. This study was supported by the High-level Key Clinical Specialty project of Guangdong Provincial Health Commission (supporting construction funds of Shenzhen) (SZGSP012), Shenzhen Science and Technology program (KCXFZ20201221173400002), Guangdong Highlevel Hospital Construction Fund Clinical Research Project of Shenzhen Children's Hospital (LCYJ2022088and LCYJ2022071), and Sanming Project of Medicine in Shenzhen (No.202411011).”

The original version of this article has been updated.

