# Supplementary material for: Body roundness index outperforms traditional obesity metrics in predicting cardiometabolic risk among children and adolescents: the EMSNGS study
Source: Front Nutr. 2026 Feb 24;13:1760511. doi: 10.3389/fnut.2026.1760511 (PMC12971936; doi:10.3389/fnut.2026.1760511)
Supplement: Supplementary file 1 [file Table_1.docx]

**Supplementary Table 1.** Binary logistic regression analysis of high BRI levels and the presence of high cardiometabolic risk.

| Group |  | B Coefficient |  | Wald |  | OR |  | 95%*CI* | | |  | *P* value |
| --- | --- | --- | --- | --- | --- | --- | --- | --- | --- | --- | --- | --- |
| BRI-boys |  |  |  |  |  |  |  |  | | |  |  |
| Q1(＜P25^th^) |  | -0.272 |  | 2.552 |  | 0.762 |  | 0.546 | | 1.064 |  | 0.11 |
| Q2(P25^th^~P50^th^) |  | Refernce | | | | | | | | | | |
| Q3(P50^th^~P75^th^) |  | 0.223 |  | 2.364 |  | 1.25 |  | 0.941 | 1.661 | |  | 0.124 |
| Q4(≥P75^th^) |  | 1.19 |  | 92.659 |  | 3.288 |  | 2.58 | 4.19 | |  | <0.01 |
| BRI-girls |  |  |  |  |  |  |  |  |  | |  |  |
| Q1(＜P25^th^) |  | -0.227 |  | 2.685 |  | 0.797 |  | 0.607 | 1.046 | |  | 0.101 |
| Q2(P25^th^~P50^th^) |  | Refernce | | | | | | | | | | |
| Q3(P50^th^~P75^th^) |  | 0.338 |  | 5.311 |  | 1.403 |  | 1.052 | 1.87 | |  | 0.021 |
| Q4(≥P75^th^) |  | 0.975 |  | 49.309 |  | 2.651 |  | 2.019 | 3.48 | |  | <0.01 |

**Note:** Logistic regression analysis was used to calculate the odds ratios of high CMRI associated with the increase in the BRI, compared to the second quantile group (Q2) and are presented in the figure. Q1 to Q4 represent the quartiles of grip strength indices: quartiles (Q1 < 25th, 25th ≤ Q2 < 50th, 50th ≤ Q3 < 75th, and Q4 ≥ 75th). High CMRI was defined as CMRI ≥ 1 (after omitting WC). All estimates are adjusted for age and pubertal stage.
